# Golgi Localized Barley *MTP8* Proteins Facilitate Mn Transport

**DOI:** 10.1371/journal.pone.0113759

**Published:** 2014-12-08

**Authors:** Pai Pedas, Michaela Schiller Stokholm, Josefine Nymark Hegelund, Anne Hald Ladegård, Jan Kofod Schjoerring, Søren Husted

**Affiliations:** Plant and Soil Science Laboratory, Department of Plant and Environmental Sciences, Faculty of Science, University of Copenhagen, Copenhagen, Denmark; Simon Fraser University, Canada

## Abstract

Many metabolic processes in plants are regulated by manganese (Mn) but limited information is available on the molecular mechanisms controlling cellular Mn homeostasis. In this study, a yeast assay was used to isolate and characterize two genes, *MTP8.1* and *MTP8.2*, which encode membrane-bound proteins belonging to the cation diffusion facilitator (CDF) family in the cereal species barley (*Hordeum vulgare*). Transient expression in onion epidermal cells showed that MTP8.1 and MTP8.2 proteins fused to the green fluorescent protein (GFP) are localized to Golgi. When heterologously expressed in yeast, MTP8.1 and MTP8.2 were found to be Mn transporters catalysing Mn efflux in a similar manner as the Golgi localized endogenous yeast protein Pmr1p. The level of *MTP8.1* transcripts in barley roots increased with external Mn supply ranging from deficiency to toxicity, while *MTP8.2* transcripts decreased under the same conditions, indicating non-overlapping functions for the two genes. In barley leaves, the expression of both *MTP8* genes declined in response to toxic Mn additions to the roots suggesting a role in ensuring proper delivery of Mn to Golgi. Based on the above we suggest that barley MTP8 proteins are involved in Mn loading to the Golgi apparatus and play a role in Mn homeostasis by delivering Mn to Mn-dependent enzymes and/or by facilitating Mn efflux via secretory vesicles. This study highlights the importance of MTP transporters in Mn homeostasis and is the first report of Golgi localized Mn^2+^ transport proteins in a monocot plant species.

## Introduction

Manganese is next to iron (Fe) the most prevalent transition metal in the earth's crust and can be found in potentially toxic concentrations in many environments. Despite the many important physiological functions of manganese (Mn) in the plant cell, the amount required for optimal growth is relatively low. However, the Mn^2+^ uptake capacity greatly exceeds this requirement [1], [2] and consequently an effective efflux system must exist to ensure homeostasis of Mn in plant tissue. The Mn^2+^ concentration in soil solution increases with decreasing soil pH and redox potential [3]. Mn toxicity is therefore a widespread phenomenon in acidic and waterlogged soils, constituting approximately 30% of the Earth's surface [4].

As an essential micronutrient, Mn has many functions in plant metabolism. In the cytosol, Mn activates the enzyme phenylalanine ammonia-lyase (PAL) of the shikimic pathway, leading to biosynthesis of various key metabolites *e.g.* the cell wall component lignin [5]. In peroxisomes and mitochondria, Mn acts as an inorganic cofactor of Mn superoxide dismutase (Mn-SOD), a protein responsible for scavenging of reactive oxygen species [6], [7]. Also, a number of glycosyl transferases localized in Golgi has been shown to have Mn-dependent activity. Some of these enzymes, such as arabinan arabinosyltransferase, xyloglucan glycosyltransferase, UDP-xylose glucuronyltransferase and arabinoxylan arabinosyltransferase, catalyse synthesis of cell wall polysaccharides [8]-[11]. Finally, Mn is also an unspecific activator of a number of different enzymes such as decarboxylases and dehydrogenases in the tricaboxylic acid cycle [3]. However, the most prominent role of Mn is its involvement in the oxygen evolving complex (OEC) of photosystem II (PSII). PSII is responsible for the photolytic dissociation of water, which releases molecular oxygen and protons while initiating the photosynthetic electron flow [12]. Due to its wide variety of essential functional properties, Mn deficiency leads to multiple visual symptoms in plants. Among these are interveinal chlorosis, necrotic spots, lower root biomass and slack leaves due to a reduction in the content of fructans and structural carbohydrates, resulting in an overall reduction in the yield potential of Mn-deficient plants [13]-[15].

Cellular Mn homeostasis in plants is regulated by processes involved in uptake, translocation, cellular sequestration and storage of Mn [16], [17]. Symplastic Mn transport is facilitated by several transport proteins. In *Arabidopsis thaliana*, the major player in Mn^2+^ uptake during conditions of Mn limitation appears to be the natural resistance-associated macrophage protein NRAMP1, which is a high-affinity Mn^2+^ transporter located in the root plasma membrane [18]. The *nramp1* mutant has both reduced biomass production and a threefold reduction in Mn shoot concentrations when compared to wild type plants. As a result, the *nramp1* mutant is sensitive towards Mn deficiency [18]. Also, the plasma membrane localized multi-specific ion transporter IRT1 from *Arabidopsis* has been shown to transport Mn^2+^ into the cell [19],[20], although the primary role of AtIRT1 is related to Fe^2+^/Fe^3+^ uptake, especially during Fe deficiency.

In the tonoplast, AtCAX2 belonging to the cation exchanger (CAX) family has the ability to transport Mn^2+^ into the vacuole. Expression studies in tobacco (*Nicotiana tabacum*) and yeast (*Saccharomyces cerevisiae*) have indicated a role for AtCAX2 in Mn^2+^ removal from the cytosol during excess Mn^2+^ conditions [21]-[24]. In seeds, the vacuolar iron transporter VIT1 from *Arabidopsis* is involved in storage of Mn and Fe, thus ensuring the availability of micronutrients during early growth of seedlings [25]. The third known vacuolar Mn^2+^ transporter, ShMTP1, has been identified in the Mn-tolerant tropical legume *Stylosanthes hamata* and is a member of the cation diffusion facilitator (CDF) transporter family. Heterologous expression of ShMTP8 in yeast or *Arabidopsis* results in increased Mn tolerance and higher cellular contents of Mn [26] (ShMTP1 has been renamed to ShMTP8 following the nomenclature for Mn-MTP proteins; [27]). Recently, the MTP8 homolog from rice has been isolated in a yeast Mn tolerance screen. OsMTP8 was found to be localized to the vacuole and it is suggested that OsMTP8 is important for Mn detoxification by sequestering Mn in the vacuole of rice plants [28]. Remobilization of vacuolar Mn in *Arabidopsis* is mediated by NRAMP3 and NRAMP4. In mesophyll cells, these proteins are likely to be involved in transport of Mn^2+^ from the vacuole to chloroplast, thus ensuring optimal OEC activity in PSII. However, neither NRAMP3 nor NRAMP4 seems to be involved in supplying vacuolar stored Mn^2+^ to the mitochondrial Mn superoxide dismutase [29], [30].

Transport of intracellular Mn^2+^ can also be mediated by P-type ATPases belonging to the subfamily P_2A_. The endoplasmic reticulum-type Ca^2+^-ATPase 1 (ECA1), from *Arabidopsis* transports Mn into ER [31]. The ECA1 homolog AtECA3 has been localized to Golgi and the growth of *eca3* mutants was severely hampered under low Mn conditions indicating a crucial role under Mn limiting conditions [32]. However, an additional study has shown that AtECA3-GFP is associated with prevacuolar compartments and AtECA3 seems to be involved in detoxification of excess Mn^2+^ since *eca3* mutants were sensitive to 50 µM Mn^2+^ [33]. Two additional genes, ECA2 and ECA4, are identified in the *Arabidopsis* genome [34], but are still not characterized. MTP11 from *Arabidopsis* was localized to the Golgi apparatus by Peiter et al [55] and they suggested that AtMTP11 is involved in Mn tolerance by loading Mn into secretory vesicles that allows Mn removal from the cell via exocytosis. A contrasting study localized AtMTP11 to the prevacuolar compartment indicating compartmentalization as the tolerance mechanism [35]. These findings indicate that AtMTP11 could be dynamically localized depending on the actual Mn status and cellular conditions of the plant tissue. Taken together, the studies conducted on Mn homeostasis show that Mn homeostasis is regulated by a highly dynamic series of transporters depending on the Mn status of the plant.

The majority of studies concerning Mn transport proteins have so far been obtained from dicotyledonous plants and solid experimental evidence regarding transport proteins controlling Mn^2+^ transport in monocotyledonous plants are lacking. In addition to OsMTP8, four plasma membrane localized transporters from monocot plant species have been identified. First, the IRT1 homologue from barley is a multi-substrate protein, which in addition to Mn also has the ability to transport Fe, Zn and Cd. Transcription of *IRT1* is induced by Fe and Mn deficiency, where both deficiencies independently lead to an increased Mn uptake capacity of the root [36]. The second Mn transport protein from monocots is the yellow stripe-like protein 2 (YSL2) from rice. OsYSL2 mediates *in vitro* transport of both Mn(II)- and Fe(III)-nicotianamine. *OsYLS2* expression is particularly pronounced in companion cells in roots, phloem cells of the vascular bundles in leaves and in developing seeds [37]. Based on the physiological performance of different rice lines with varying expression levels of *OsYSL2*, the encoded transporter has been suggested to operate in Fe and Mn translocation, especially to shoots and the endosperm of developing grains [38]. The YSL2 homologue OsYSL6 is involved in Mn translocation between the apoplast and symplast in leaves, a process which could affect the plants tolerance towards Mn toxicity [39]. Finally, in rice OsNRAMP3 is shown to regulate Mn shoot distribution depending on environmental conditions [40], [41]. The OsNRAMP3 protein is constitutively expressed except during Mn excess conditions, where it is internalized in vesicles and rapidly degraded. The vascular-bundle specific localization in nodes ensures that Mn follows the transpiration stream to the youngest leaves during low and moderate Mn conditions, whereas during Mn excess Mn is translocated to old leaves and thereby preventing Mn toxicity in the young shoot tissue.

In the present study we have used yeast as a tool to identify and characterize two novel barley genes, *MTP8.1* and *MTP8.2*, encoding Mn^2+^ transport proteins. Root transcripts of *MTP8.1* and *MTP8.2* show contrasting responses towards external concentrations of Mn, suggesting that the two proteins are transcriptionally regulated depending on the Mn nutritional status of the plant. Both MTP8.1 and MTP8.2 proteins fused to the green fluorescent protein (GFP) were localized to Golgi. This is the first report of Golgi localized Mn^2+^ transport proteins that are involved in intracellular Mn homeostasis of a monocot plant species.

## Materials and Methods

### Functional complementation in yeast

Wild type (WT) yeast (*Saccharomyces cerevisiae*) strain (BY4741) and 7 deletion mutants in the same genetic background were used in this study (Table 1). The yeast strains were transformed with either *MTP8.1*, *MTP8.2* expressed from pFL61 [36] or the empty vector. Transformants were grown on synthetic minimal media composed of 2% glucose (SD), 50 mM succinic acid/Tris base, pH 5.5, 0.7% yeast nitrogen base w/o amino acids (Difco), 0.3% of appropriate amino acids (histidine (H), leucine (L), methionine (M)). The presence of URA3 on pFL61 was used as auxotroph marker for uracil synthesis. For solid medium 2% agar was added [42]. Media deprived of Ca^2+^ were obtained using the chelators ethylene glycol-*bis*-β-aminoethylether-*N*,*N*,*N′*,*N′*-tetreacetic acid (EGTA, Sigma) or (2,2-bis(o-aminophenoxy)ethane-N,N,N′,N′-tetra acetic acid) (BAPTA, Sigma). The metal concentrations used as supplements in yeast media are presented in Table 1. Plates were incubated at 30°C for 3-5 days. All growth assays were repeated three times with independent yeast transformants.

**Table 1:** List of yeast (*Saccharomyces cerevisiae*) strains used in this study.

Mutant	Background	Mating type	Genotype	Selectivity tested	Concentration	Localization of knockout protein	Reference
*pmr1Δ*	BY4741	**a**	his3Δ1 leu2Δ0 met15Δ0 ura3Δ0YGL167c::kanMX4	Mn^2+^	0, 1, 2 mM Mn^2+^	Golgi	Euroscarf Y04534
*pmr1Δ*	BY4741	**a**	his3Δ1 leu2Δ0 met15Δ0 ura3Δ0YGL167c::kanMX4	Ca^2+^	0, 500, 750, 1000, 1500 µMBAPTA^I^ and 5, 10, 15, 20mM EGTA^II^	Golgi	Euroscarf Y04534
*ycf1Δ*	BY4741	**a**	his3Δ1 leu2Δ0 met15Δ0 ura3Δ0YDR135c::kanMX4	Cd^2+^	0, 10, 20, 40, 60, 100, 120 µM Cd^2+^	Vacuolar membrane	Euroscarf Y04069
*cot1Δ*	BY4741	**a**	his3Δ1 leu2Δ0 met15Δ0 ura3Δ0 YOR316c::kanMX4	Co^2+^	0, 1, 1.5, 2 mM Co^2+^	Vacuolar membrane	Euroscarf Y01613
*ace1Δ*	BY4741	**a**	his3Δ1 leu2Δ0 met15Δ0 ura3Δ0YGL166w::kanMX4	Cu^2+^	50, 75, 100, 125, 150, 175,200 µM Cu^2+^	Transcription factor	Euroscarf Y04533
*ccc1Δ* ^III^	BY4741	**a**	his3Δ1 leu2Δ0 met15Δ0 ura3Δ0YLR220w::kanMX4	Fe^2+^/Fe^3+^	0, 2.5, 3, 4, 4.5, 5 mMFe^2+^/Fe^3+^	Vacuolar membrane	Euroscarf Y04169
*smf1Δ*	BY4741	**a**	his3Δ1 leu2Δ0 met15Δ0 ura3Δ0YOL122c::kanMX4	Ni^2+^	0,1,1.25, 1.5 mM Ni^2+^	Plasma membrane	Euroscarf Y06272
*zrc1cot1Δ(CK1)*	BY4741/BY4742	**a**	his3Δ1 leu2Δ0 met15Δ0 ura3Δ0YOR316c::kanMX4; YMR243c::natMX	Zn^2+^	0, 1, 2 mM Zn^2+^	Vacuolar membrane	Mills *et al.*, 2005
WT	BY4741	**a**	his3Δ1 leu2Δ0 met15Δ0 ura3Δ0				Euroscarf Y00000

I: BAPTA (1,2-bis(o-aminophenoxy)ethane-N,N,N',N'-tetra acetic acid)

II: EGTA (ethylene glycol-*bis*-β-aminoethylenether-N,N,N′,N′-tetraacetic acid)

III: The YCp-vector His 3 marker (Stratagene PRS-413) were transformed into the *ccc1* yeast strain achieving a yeast strain sensitive on high Fe^2+^/Fe^3+^ levels [25].

Barley MTP8 proteins were tested for their metal ion selectivity in different yeast mutants at different metal ion concentrations as given below. The accessions numbers for each strain obtained from Euroscarf are given (www.euroscarf.de) [60].

### Identification of Mn transporter proteins

The yeast strain *pmr1Δ* was transformed by electroporation with a cDNA library from barley roots inserted into the vector pFL61 as previously described [36]. Transformants were selected on minimal media lacking uracil, harvested from the solid media, pooled, and resuspended in glycerol. From this pool, yeast aliquots were re-grown on medium with 2 mM Mn^2+^ to select for yeast cells complementing the Mn sensitivity of the *pmr1Δ* mutant. Plasmids were extracted from colonies complementing *pmr1Δ* [43] and amplified in *Escherichia coli* by standard procedures. Plasmids complementing *pmr1Δ* after re-transformation were sequenced and the barley genes were identified from similarity searches using BLAST analysis.

### Yeast growth assay

Transformed *pmr1Δ* cells were cultured in 2 ml minimal media overnight at 30°C. The next day, 120 ml fresh medium was added and cells were re-incubated O/N to obtain a healthy yeast culture. The following day, all strains were inoculated in triplicates at seven different Mn^2+^ concentrations (0, 0.1, 0.25, 0.5, 1, 2, 5 mM) in 50 ml SD-HLM media at OD_600_  = 0.02.and grown for 46 hours at 30°C.

### Elemental profile of MTP8 transformed yeast cells

Wild type and *pmr1Δ* transformed cells were grown overnight in 50 ml synthetic media. The next day cells were washed twice with ultra-pure H_2_O and re-suspended in 40 ml minimal media in polypropylene bottles at initial OD_600_ values of 0.06. The assay was performed with three replicates using two Mn^2+^ concentrations (2.4 and 100 µM). The yeast cells were grown to mid-exponential phase (17 hours at 150 rpm). The cells were again washed twice with ultra-pure H_2_O and then transferred to acid washed test tubes and freeze dried in a freeze drier (Christ Alpha 1-4, Martin Christ Gmbh).

The dry and weighed yeast samples were digested in 5 ml 70% HNO_3_ (J.T. Baker 9598) using a microwave oven (Multiwave 3000, software version 1.24, Anton Paar GmbH). Samples were diluted to 7% HNO_3_ and analyzed using Inductively Coupled Plasma Optical Emission Spectroscopy (ICP-OES, Optima 5300 DV; Perkin-Elmer) with the following wavelengths; 315.881A (Ca), 228.797A (Cd), 228.610A (Co), 324.750R (Cu), 238.199A (Fe), 766.473R (K), 279.072A (Mg), 257.606A (Mn), 214.909R (P), 181.971R (S), and 213.852A (Zn) nm. The accuracy and precision of the ICP-OES measurements were verified by including a certified reference material (Apple leaf, standard reference material 1515, National Institute of Standards and Technology). Data was accepted if the accuracy was above 90% of the standard reference material value for a given element.

### Plant material

Seeds of *Hordeum vulgare* L. cv. ‘Vanessa’ were germinated at 21°C in vermiculite. After 5 d, uniform seedlings were selected and transferred to light-impermeable 4 L black cultivation units, each holding four plants. The units were filled with a chelate-buffered solution prepared in double ionized water with nutrient concentrations, except for Mn, as specified by Pedas et al. (2005). Four treatments were applied with different amounts of Mn (viz. 0 µg Mn (deficiency), 7.33 µg Mn (control), 110 µg Mn (toxicity I - Mn_tox1_), and 586 µg Mn (toxicity II - Mn_tox2_)) added to each cultivation unit three times per week in the form of MnCl_2_. The pH was kept at 6.0 by using 0.5 mM MES-TRIS pH 6.0. The plants were grown for 39 days in a controlled growth chamber with a 250 to 280 µmol m^-2^ s^-1^ photon flux density, 75% to 80% humidity, and a 20°C/15°C (16/8) day/night temperature regime.

### Determination of trace element concentrations in plant material

Multi-elemental analyses by ICP-MS of leaf tissue were performed and compared with critical threshold limits found in [44]. Prior to analysis, plant samples were freeze dried (Christ Alpha 2-4; Martin Christ GmbH)) and digested using ultra-pure acids as previously described [1].

### Analysis of gene expression in planta

Total RNA was isolated from root and shoot samples using the Fast RNA Pro Green Kit (MP Biomedicals, Solon, OH, USA) followed by TURBO DNase treatment (Applied Biosystems). RNA was converted to cDNA with M-MuLV Reverse Transcriptase (New England BioLabs), oligo-(dT) and random hexamer primers. The cDNA was diluted 5 times and normalized by further dilution to get the same DNA concentration in all samples. For quantification of *MTP8*.*1*, *MTP8*.*2* and *IRT1* gene expression, *ACTIN* was used as reference gene for normalization and cDNA was amplified by RT-qPCR using a Mx3000P Real-Time PCR System (Stratagene) in a total volume of 20 µl per reaction including: 1 µl normalized cDNA, 0.3 µM gene specific forward and reverse primers (Table 2), 1× DyNAmo Flash Master Mix and 0.4× ROX Passive Reference dye from DyNAmo Flash SYBER Green qPCR Kit (Finnzymes). The qPCR programme was set as follows: One cycle at 95°C for 7 minutes, followed by 40 cycles of 95°C for 10 seconds and 60°C for 30 seconds. A dissociation curve to check specificity of the amplified products was performed in the end of each programme with one cycle at 95°C for 1 min, 60°C for 30 seconds, ramping up to 95°C, followed by 1 minute at 95°C. Three biological replicates of each treatment were included and each reaction was performed in duplicate. The Pfaffl equation [45] was applied to calculate the relative expression levels. A standard curve was performed for each primer pair prior to RT-qPCR analysis in order to determine the amplification efficiency required for the Pfaffl equation.

**Table 2:** List of primers used for RT-qPCR and GFP fusion constructs.

Gene	Forward	Reverse	Product size
**RT-qPCR**			
*ACTIN*	5′-TCGCTCCACCTGAGAGGAAG-3′	5′-GCTAGGATGGACCCTCCGAT-3′	52 bp
*IRT1*	5′-CGTCTTCTTCTTCT-3′	5′-GGGGCTGTTGTCCTT-3′	82 bp
*MTP8.1*	5′-GTGTGACCACAAGCCAGAGC-3′	5′- GCATGTTGTCCACGGTGAAT-3′	89 bp
*MTP8.2*	5′- CTTCGTGGAGGTTGACATCG -3′	5′- GGCAGCTTCTCGATCCTCTC-3′	96 bp
**GFP**			
*MTP8.1*	5′-ACAAGTTTGTACAAAAAAGCAGGC-3′	5′-GACCACTTTGTACAAGAAAGCTGGGTCTGCAGGCTGGCTGCTAG-3′	1233 bp
*MTP8.2*	5′-ACAAGTTTGTACAAAAAAGCAGGC-3′	5′-GACCACTTTGTACAAGAAAGCTGGGTCTGCGGGCTCGGTCGC-3′	1263 bp

### Subcellular localization of MTP8-GFP fusion proteins in onion epidermal cells

A PCR-based cloning strategy was used to generate *MTP8* gene sequences without stop codon for C-terminal fusions to the *GFP* gene. The cloning reaction used forward primers specific to the gateway cassette in pFL61 and reverse primers specific to C-termini of *MTP8.1* and *MTP8.2*, respectively (Table 2) The PCR products were amplified using LA Taq (Takara), cloned using the gateway technique (Invitrogen), and sequenced by Eurofins MWG-Biotech, Germany. Correct clones were subsequently sub-cloned via the gateway technique (Invitrogen) to the *p2GWF7* vector, consisting of the *CaMV35S* promoter, a *NOS* terminator, and the ORF of *GFP* [46]. *p2GWF7-MTP8.1-GFP* and *p2GWF7-MTP8.2-GFP* were used to transiently express MTP8.1- and MTP8.2-GFP fusion proteins in onion (*Allium cepa*) epidermal cells. Transient expression and localization were done as described by [47] with a few modifications. Briefly, 1×1 cm^2^ of onion bulb scales were placed on agar (1x Murashige and Skoog medium, 0.7% agar, and 3% Suc, pH 5.8) with the inner epidermis facing up. They were bombarded using the PDS-1000/He biolistic particle delivery system (Bio-Rad). Approximately 6 µg of expression vector was coated onto 1.5 mg of 1.6-*µ*m gold particles and transferred into the cells. After bombardment, petri dishes containing the onion bulb squares were placed in the dark at room temperature for 18 to 24 h. The epidermis was then peeled and transformed cells were visualized using a Leica TCS SP2/MP confocal laser scanning microscope (Leica Microsystems). As a positive control for Golgi visualization, GONST-YFP proteins were co-localized with the MTP8-GFP proteins [48]. Excitation for GFP and YFP was done at 488 nm, and the emission spectra were recorded between 495 and 510 for GFP (green channel) and 530 and 545 nm for YFP (red channel). Sequential scanning between lines was used to follow both fluorescent signals at once. No fluorescence bleed-through was detected under our experimental conditions (data not shown). The co-localization analyses with GONST1-YFP were conducted with minimum three independent transient experiments. A quantitative co-localization analysis was performed using Fiji (ImageJ 1.49g) with a colocalization threshold plug-in (http://imagej.nih.gov/ij/index.html).

### Accession numbers

The GenBank accession numbers for the sequences described in this article are as follows: *Arabidopsis thaliana* (AtMTP8: NP_191365; AtMTP9: NP_178070; AtMTP10: NP_173081; AtMTP11: NP_181477), *Stylosanthes hamata* (ShMTP8: AY181256; ShMTP9: AY181257; ShMTP10: AY181258; ShMTP11: AY181259), Oryza sativa (Os02g53490/OsMTP8; Os03g12530; Os01g03910; Os01g03914; Os05g38670; Os01g62070) and *Hordeum vulgare* (MTP8.1: JX051321; MTP8.2: JX051322; IRT1: EU545802; ACTIN: TC131547).

## Results

### Isolation of MTP8 genes from a yeast screen for barley Mn tolerance genes

**Figure 1:**
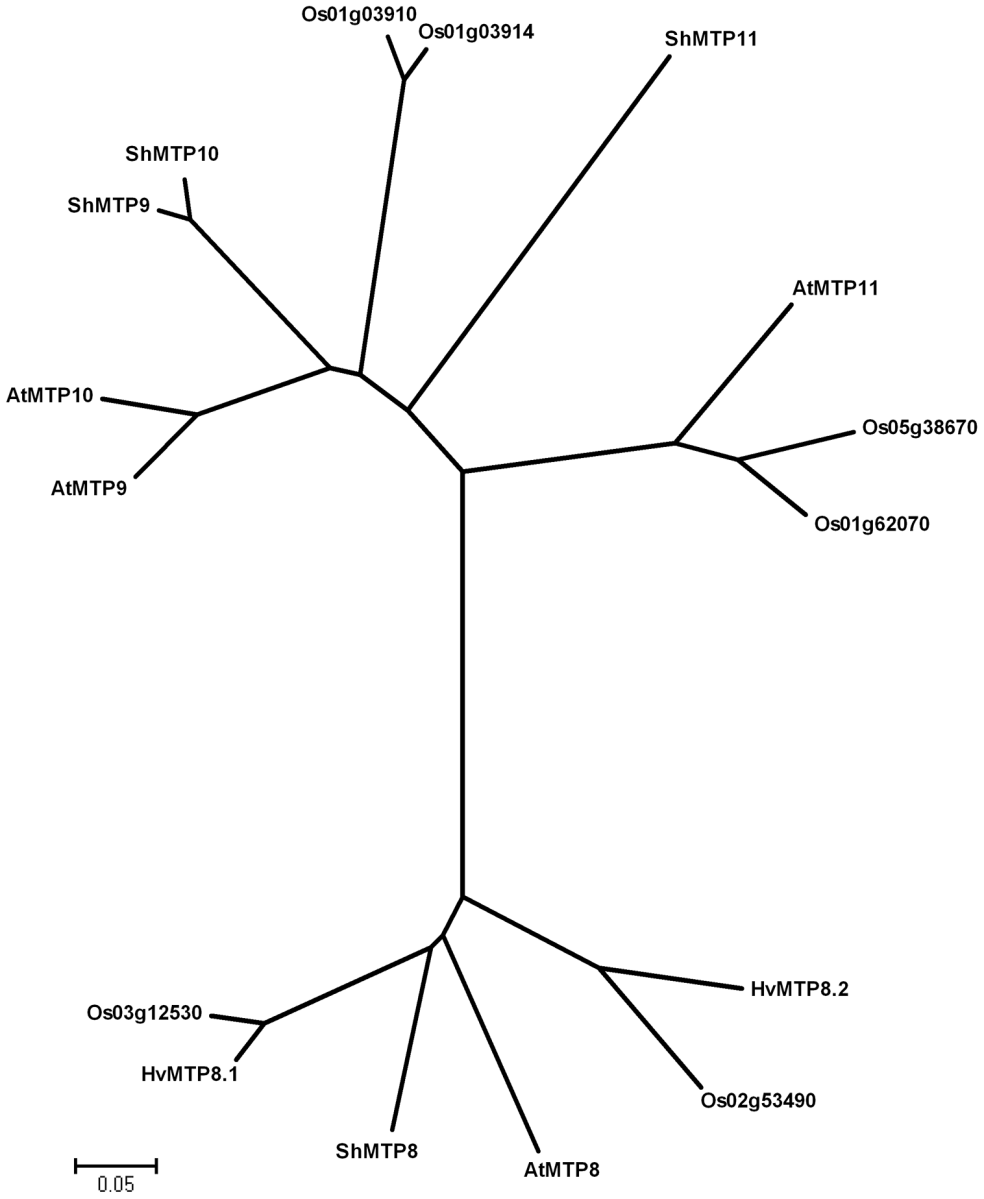
Phylogenetic analysis of 16 MTP protein sequences from *Hordeum vulgare* (Hv), *Oryza sativa* (Os), *Arabidopsis thaliana* (At) and *Stylosanthes hamata* (Sh). Alignment of full-length sequences was done as described for S1 Figure. The phylogenetic tree was drawn with the MEGA version 4.0 (http://www.megasoftware.net) [59]. Numbers represent bootstrap values for 1000 trees.

To identify transport proteins involved in intracellular Mn^2+^ transport, we employed a yeast screen using the *pmr1Δ* mutant, which is deleted in *PMR1* encoding a Ca^2+^/Mn^2+^-specific Golgi localized P-type ATPase [49], [50]. The *pmr1Δ* cells were transformed with a cDNA library prepared from barley root RNA. Complementing yeast transformants were selected on media containing 2 mM Mn, a condition which is toxic to *pmr1Δ* yeast cells [51]. Of 150 analyzed yeast transformants, 95% of the colonies were transformed with the same barley cDNA clone, 4% contained a second barley clone and 1% were false positives. The sequences of the two barley cDNA clones showed similarity to cation diffusion facilitator (CDF) family genes. The CDF family is a ubiquitous family of heavy metal transporters, found in all three kingdoms of life: Archaea, Eubacteria and Eukaryotes. All known CDF substrates are divalent cations (Me^2+^) and most of the characterized transporters are Me^2+^/H^+^ (K^+^) antiporters involved in efflux of the metal ion from the cytoplasm to the outside of the cell or into subcellular organelles (Montanini et al., 2007). Using bioinformatics, the barley MTP8 proteins were found to cluster within the MTP-subgroup of Mn specific transporters (data not shown). A phylogenetic analysis of protein data from Mn specific MTP plant proteins originating from *Arabidopsis*, *Stylosanthes hamata*, rice and barley revealed that the barley MTP proteins identified here belong to a subgroup, which contains MTP8-like proteins (Fig. 1). The barley proteins were consequently named *MTP8.1* and *MTP8.2*. The closest relatives to the MTP8 proteins were two uncharacterized rice proteins Os03g12530 and Os02g53490, showing 92.4 and 69.3% identity to MTP8.1, and 70 and 80.7% identity to MTP8.2, respectively.

The barley MTP8 proteins both include the conserved glycine and aspartate residues as well as the N-terminal CDF signature sequence [27], [52] (S1 Figure). MTP8.1 and MTP8.2 consist of 400 and 410 amino acids, respectively, corresponding to predicted molecular masses of 44.8 kDa and 45.7 kDa. Secondary structure predictions suggest five transmembrane (TM) domains in MTP8.1, whereas MTP8.2 contains four TM domains.

### Screen for MTP8 metal selectivity in yeast

**Figure 2:**
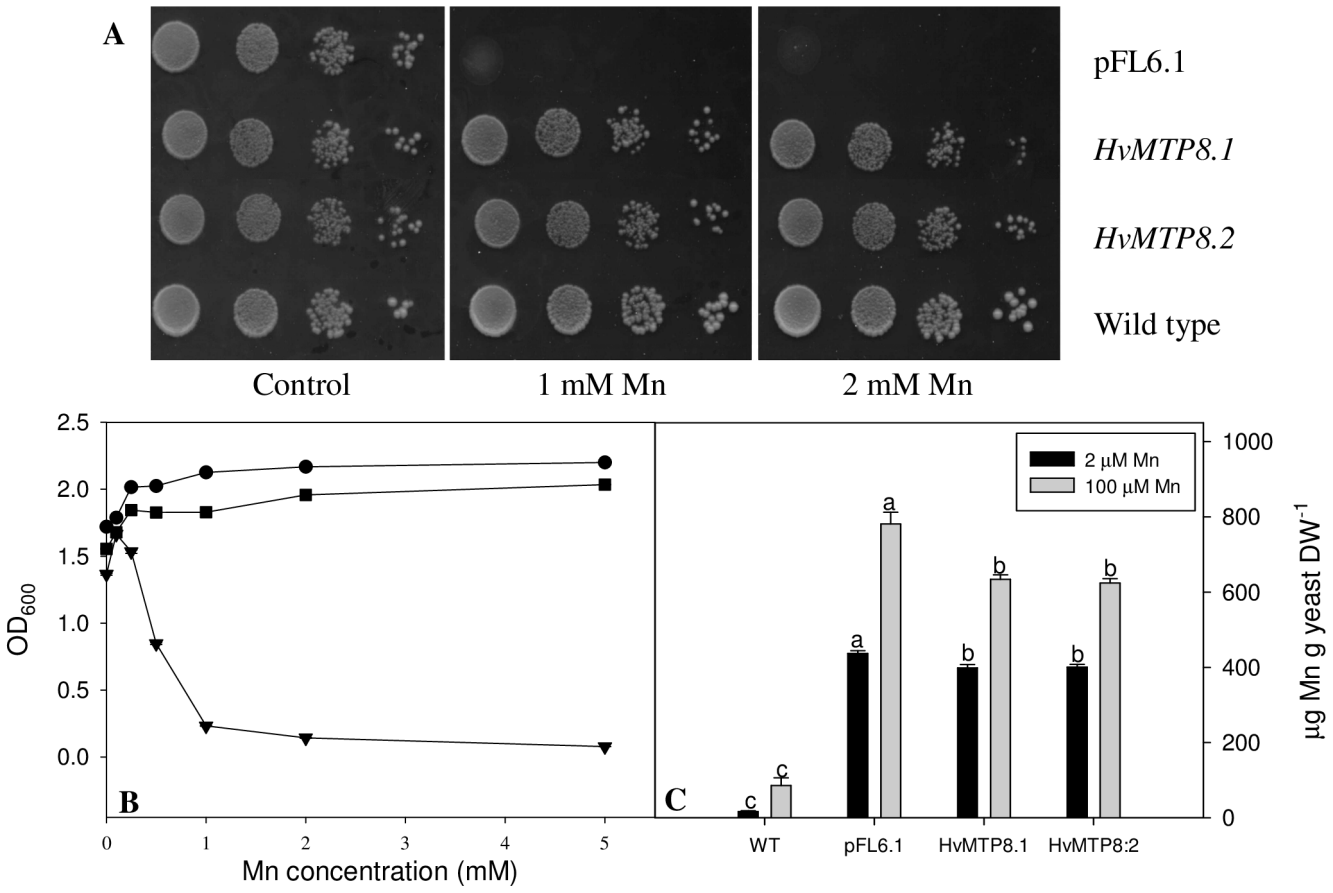
Effect of *MTP8.1* and *MTP8.2* expression on Mn^2+^ tolerance and Mn accumulation in the yeast mutant *pmr1Δ*. Serial dilutions (1.0, 0.1 and 0.01 OD_600_) of *pmr1* cells transformed with *MTP8.1*, *MTP8.2* or empty vector (pFL6.1) were spotted on SD selective media with increased Mn content (A). Plates were incubated for 3-5 days at 30°C. The results are from one representative experiment out of three independent yeast transformations. Liquid yeast growth assay with *MTP8.1* (circle), *MTP8.2* (square) and pFL6.1 (triangle) transformed *pmr1Δ* cells grown with increasing Mn concentrations over 48 hours, data are the means ± SE of three independent yeast samples (B). Mn accumulation in *pmr1Δ cells* determined by ICP-OES analysis, cells were cultivated for 17 h with either 2.4 µM (black bars) or 100 µM (grey bars) Mn^2+^ in medium (C). Data are the means ± SE of three independent yeast samples. Values with the same letter within the same treatment are not significantly different (P>0.05).

*MTP8.1* and *MTP8.2* complemented the Mn sensitivity of *pmr1Δ* cells when cultured on media with high Mn concentrations (Fig. 2A). The *pmr1Δ* mutant was also unable to grown on Ca depleted media because the Pmr1 protein is essential for loading of Ca into the ER and Golgi apparatus [49], [50]. The barley MTP8 proteins were not able to complement this phenotype (data not shown), suggesting that the MTP8 proteins are selective for Mn over Ca. Expression of MTP8 genes could not rescue numerous other yeast mutants defective in metal transport across the tonoplast of Zn and Co (*cot1Δ*, *zrc1cot1Δ*); Fe (*ccc1Δ*) or Cd (*ycf1Δ*). Also, no beneficial effects of MTP8 expression were found in *smf1Δ* or *ace1Δ* mutants defective in controlling Ni and Cu homeostasis, respectively (data not shown). The applied growth conditions for yeast complementation assays are presented in Table 1. These results clearly indicate that MTP8.1 and MTP8.2 has a preference for Mn as the transported ion.

### MTP8 proteins confer Mn tolerance to pmr1 via a Mn efflux mechanism

In liquid cultures of yeast *pmr1Δ* cells, both MTP8.1 and MTP8.2 resulted in high levels of Mn tolerance (Fig. 2B). The growth rates for *pmr1Δ* expressing either of the *MTP8* homologs saturated at Mn concentrations above 1 mM and were unaffected by concentrations up to 5 mM Mn. In contrast, the growth rate of the control cells was severely reduced by Mn concentrations above 0.5 mM and growth almost ceased at 5 mM Mn.

To determine the elemental changes resulting from MTP8 expression, Inductively Coupled Plasma-Mass Spectrometry (ICP-MS) was performed on cells grown in normal media (2.4 µM Mn) or enriched with 100 µM Mn (Fig. 2C). The external Mn concentration of 100 µM did not restrict growth of either wild type or *pmr1Δ* cells due to Mn toxicity, thereby ensuring a minimum regulation of secondary toxicity affects including yeast endogenous Mn transporters. Elemental analysis showed that deletion of *PMR1* by itself caused a 25-fold increase in the cellular Mn contents relative to the wild type. Mn concentrations in *pmr1Δ* cells expressing *MTP8.1* or *MTP8.2* were reduced by 20% when compared to *pmr1Δ* containing the empty vector (Fig. 2C). No other changes in elemental composition occurred between empty vector and *MTP8* transformed yeast cells (S2 Figure).

### Barley MTP8 proteins are targeted to the Golgi apparatus

**Figure 3:**
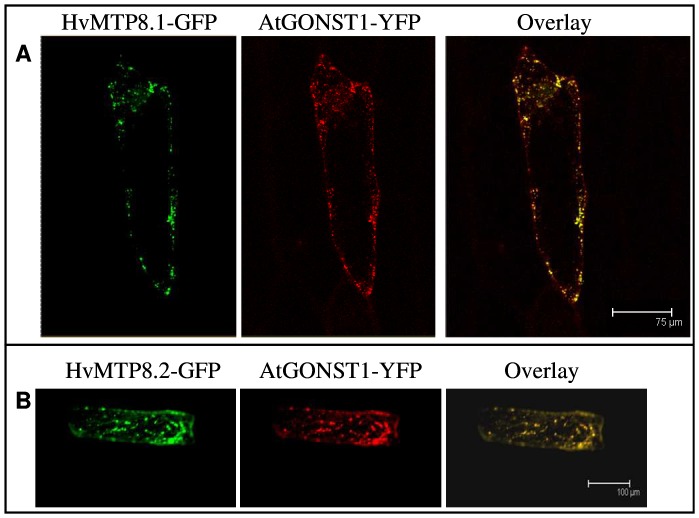
C-terminal MTP8-GFP fusion proteins localizes to the Golgi apparatus. As a control, GONST1-YFP were transiently co-expressed with MTP8.1-GFP and MTP8.2-GFP in onion epidermal cells (A and B), respectively. The right panels show co-localizations of GONST1-YFP with each MTP8-GFP protein. Scale bars were 75 and 100 µm for A and B, respectively.

To clarify the mechanism of Mn tolerance in yeast induced by MTP8.1 and MTP8.2, C-terminal fusions with green fluorescent protein (GFP) were transiently expressed in onion epidermal cells under control of the *CaMV35S* promoter. Both MTP8.1- and MTP8.2-GFP fusion proteins resulted in a punctated pattern of GFP-derived fluorescence, a highly characteristic feature of the Golgi apparatus (Fig. 3A and B, left panel). The MTP8-GFP localizations to Golgi were supported by co-expression of the Golgi-localized marker protein GONST1 fused to YFP [48] (Fig. 3A and B, right panel). A co-localization analysis was performed resulting in correlation coefficients of 0.81±0.03 and 0.85±0.05 for HvMTP8.1 and HvMTP8.2, respectively.

### Regulation of MTP8.1 and MTP8.2 in response to plant Mn status

**Figure 4:**
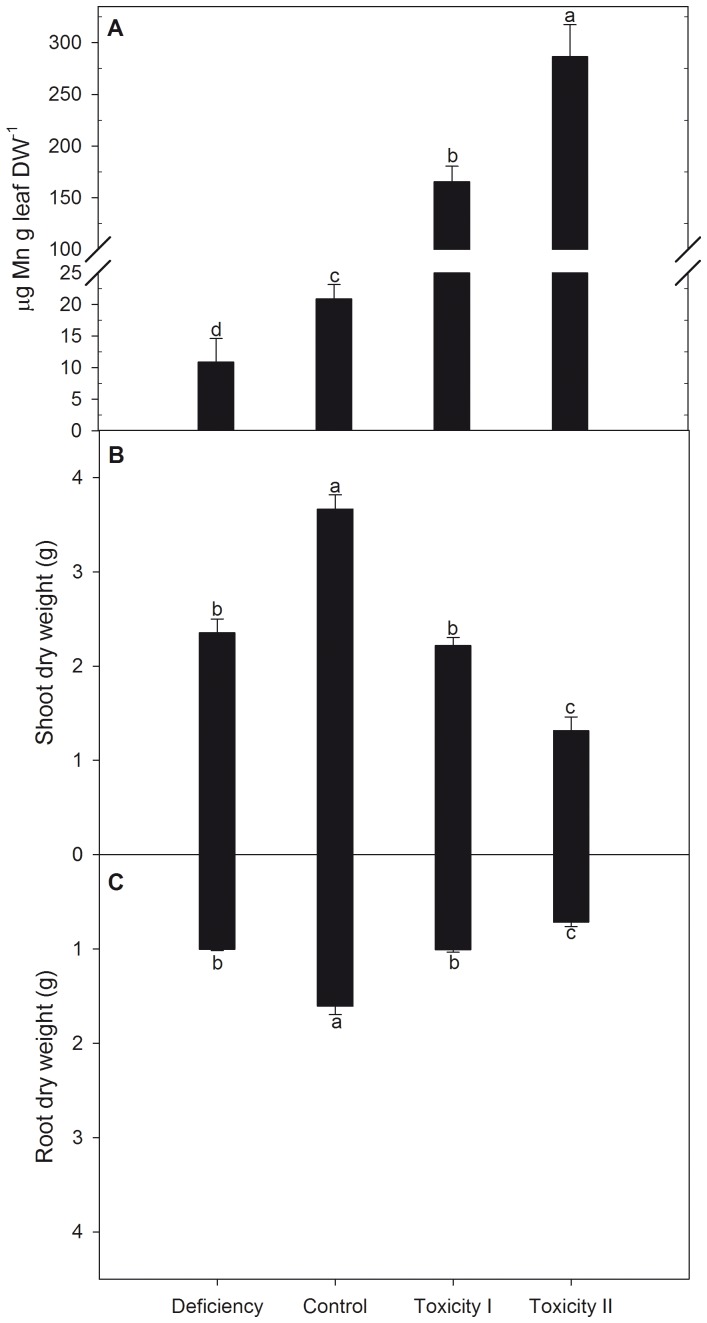
The effects of insufficient, control or two increasing Mn toxicity levels on Mn accumulation in the youngest fully developed leaves (A), shoot dry weight (B) and root dry weight (C) of barley plants. Data are means ± SE (*n* = 3). Values with the same letter between treatments are not significantly different (P>0.05).

**Figure 5:**
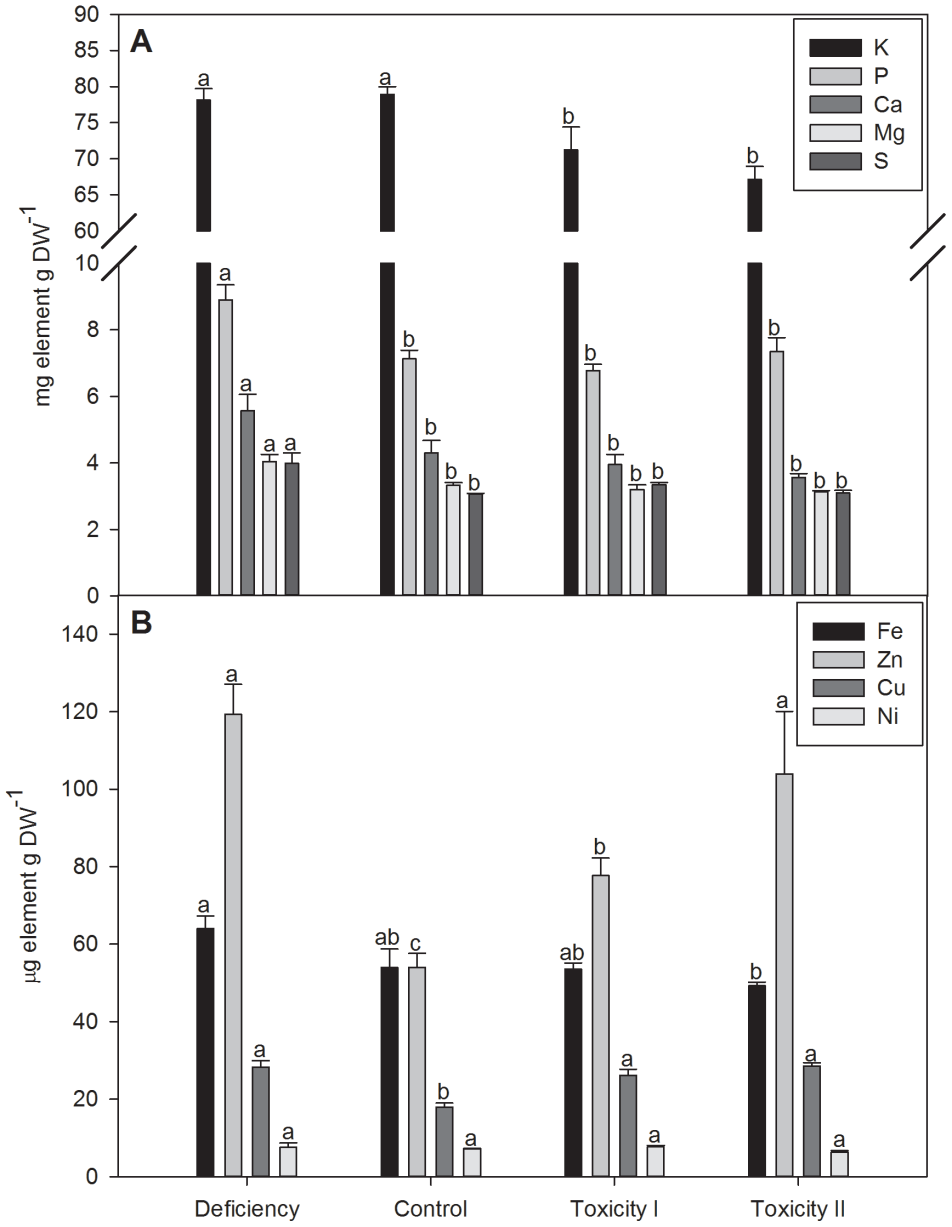
Elemental analysis of the youngest leaves of barley plants grown with insufficient, control or two increasing Mn toxicity levels. The concentrations of five macro- (A) and four micronutrients (B) were determined in the youngest leaves. Data are means ± SE (*n* = 3). Values with the same letter between treatments are not significantly different (P>0.05).

To characterize the *in planta* expression of *MTP8.1* and *MTP8.2*, barley was cultivated in hydroponics with either insufficient, adequate (control) or excessive Mn supplies. The insufficient treatment reduced leaf Mn concentrations to 10 µg Mn g^-1^ DW which is 50% lower than in the control (Fig. 4A). Excessive Mn supply (treatments Mn_tox1_ and Mn_tox2_) resulted in foliar Mn concentrations between 170 and 280 µg Mn g^-1^ DW accompanied by typical Mn toxicity symptoms such as chlorosis and necrotic brown spots (data not shown). The biomass production of root and shoot tissues decreased in response to both insufficient and excessive Mn supplies (Fig. 4B and 4C) and Mn toxicity resulted in 20% increase in leaf dry matter content (Table 3). The altered biomass production was accompanied with marked changes in the elemental profile of the youngest leaves (Fig. 5).

**Table 3:** Leaf % dry weight of barley plants grown with varying Mn supplies. Data are means ± SE (n = 3).

Treatment	Leaf % dry weight
Deficiency	9.86±0.11^b^
Control	9.77±0.09^b^
Toxicity I	11.86±0.39^a^
Toxicity II	12.46±0.56^a^

Values with the same superscript letter were not significantly different between treatments (P>0.05).

**Figure 6:**
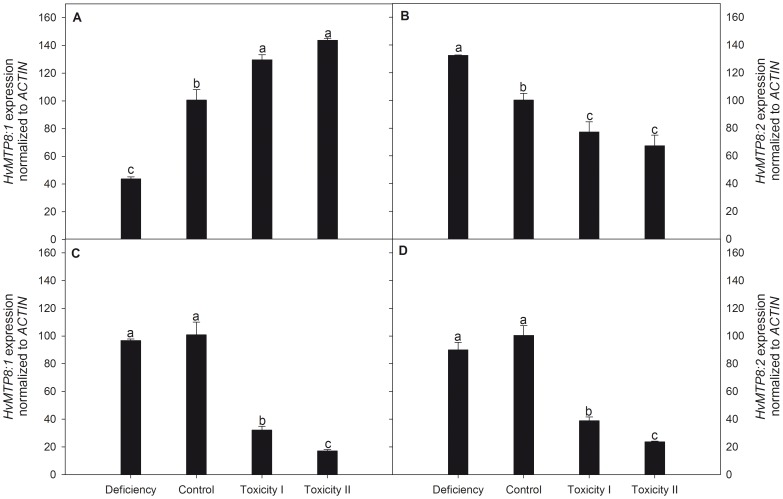
Quantitative expression analysis of *MTP8* genes in barley plants grown with insufficient, control or two increasing Mn toxicity levels. The expression level of *MTP8.1* (A and C) and *MTP8.2* (B and D) were determined in root (A and B) and shoot tissue (C and D). Data are normalized with respect to the expression of the reference gene *ACTIN*. Furthermore the relative changes in *MTP8* expression levels are indexed to barley plants grown under control conditions. Data represent means (n = 3) ± SE. Values with the same letter between treatments for each *MTP* gene and plant organ are not significantly different (P>0.05).

To determine if *MTP8* transcripts fluctuate in response to changes in Mn concentrations of the root environment, quantitative reverse transcription PCR (RT-qPCR) was used on both root and shoot tissues. In roots, *MTP8.1* and *MTP8.2* transcripts were inversely regulated (Fig. 6A and B). The *MTP8.1* transcript level increased with Mn supply ranging from deficiency to toxicity. In contrast, *MTP8.2* expression showed a decrease as the external Mn concentration increased. In Mn deficient plants, *MTP8.1* transcripts were down-regulated to 40% of the control level, whereas at the Mn_tox2_ supply transcript levels rose to 140% of that obtained in plants grown at the control settings (Fig. 6A). For *MTP8.2* transcripts, Mn deficiency increased to levels 40% higher than the control whereas transcript levels declined to 67% of the control at Mn_tox2_ supply (Fig. 6B). *MTP8.1* and *MTP8.2* transcripts did not respond to Mn deficiency in shoots, whereas toxic supply of Mn resulted in a 3-4 fold down-regulation of both *MTP8* genes in this organ (Fig. 6C and D).

**Figure 7:**
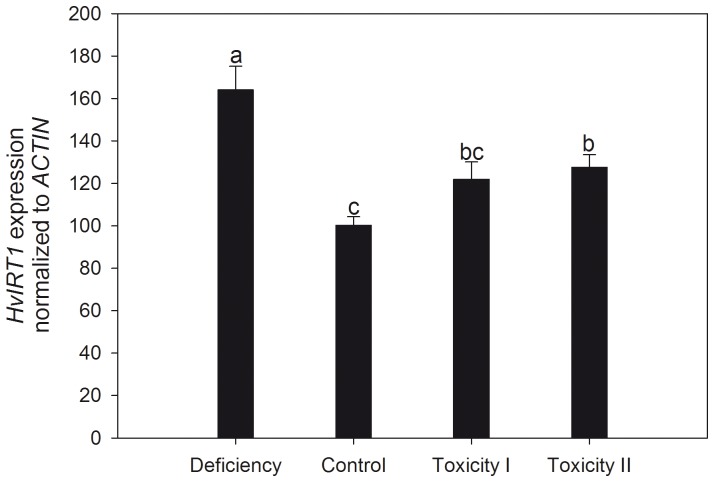
Quantitative expression analysis of *IRT1* in root tissue of barley plants grown with insufficient, control or two increasing Mn toxicity levels. Data are normalized with respect to the expression of the reference gene *ACTIN*. Furthermore the relative change in *IRT1* expression is indexed to barley plants grown under control conditions. Data represent means ± SE (n = 3). Values with the same letter between treatments are not significantly different (P>0.05).

The transcript level of the broad specific metal ion transporter IRT1, which is localized in the plasma membrane of root cells with a potential role in metal uptake, was 60% up-regulated during Mn deficiency (Fig. 7). The *IRT1* transcript levels also increased with up to 25% at toxic Mn supplies, indicating that transcriptional down-regulation of IRT1-mediated Mn uptake is not a Mn detoxification mechanism. The increased *IRT1* expression level may also be related to declining Fe status since the Fe concentration decreased roughly 20% with increasing Mn supply (Fig. 5B). Foliar Zn and Cu concentrations were lowest in control plants and increased almost 2-fold in response to both insufficient and excessive Mn supplies.

## Discussion

number of transport proteins with a putative role in cellular detoxification of excess Mn have previously been characterised in dicot plant species [26], [53]. However, similar information is currently not available for monocot species and consequently we conducted a barley cDNA library screen [36] expressed in the Mn sensitive yeast strain *pmr1Δ*. This resulted in identification of two genes encoding proteins involved in Mn tolerance when expressed in yeast. The encoded proteins were both homologous to the previously characterized MTP proteins OsMTP8 from rice [28], ShMTP8 from *Stylosanthes* [26] and AtMTP11 from *Arabidopsis* [53] (Fig. 1). The barley MTP8 proteins alleviated the growth defect of *pmr1Δ* caused by Mn toxicity (Fig. 2A), but not growth defects in a number of other yeast mutant strains related to defective Fe, Co, Zn, Ni, Cd, Ca or Cu transport (data not shown, Table 1). In addition, yeast strains expressing *MTP8* genes were found to be specifically altered in Mn content, while all other divalent metals were unchanged (Fig. 2C and S2 Figure), thus showing a preference for Mn by the MTP8.1 and MTP8.2 proteins.

Cellular tolerance to excess Mn concentrations could either involve compartmentalization or export out of the cell. Analysis of the Mn accumulation in *pmr1Δ* yeast cells expressing barley MTP8 proteins showed that these proteins are involved in Mn efflux, as *MTP8 pmr1Δ* cells had a 20% reduction in their Mn contents when compared to the control (Fig. 2C). This reduction is relatively small compared to the 25 fold reduction in Mn content caused by the presence of Pmr1p in wild type yeast compared to *pmr1Δ* mutant. However, the Mn cellular homeostasis in yeast is tightly controlled [54]. In this heterologous expression system it is therefore unlikely that a plant Mn transport protein would be able to interact with the endogenous homeostatic partners in the same manner as the native Mn transporter. Nevertheless, expression of the MTP8 proteins enabled the yeast mutant to maintain optimal growth even at 5 mM Mn in the growth medium. The elemental profile of yeast cells grown at these two non-toxic Mn concentrations, with a minimum regulation of endogenous Mn homeostasis, strongly suggests that the tolerance mechanism is Mn efflux. The reduction in Mn content may be due to the MTP8 proteins being localized in the plasma membrane and/or Golgi apparatus. Previously, the Zn transporter TgMTP1 has been suggested to have a role in Zn tolerance due to its localization to the plasma membrane [55]. Furthermore, the Mn specific AtMTP11 protein has been suggested to have a role in Mn efflux due to its localization in the Golgi apparatus, where it delivers Mn into secretory vesicles directed to exocytosis [53]. The cellular localization of the MTP8 proteins *in planta* were clarified using transiently expressed GFP fusion proteins in onion epidermal cells. Transient expression of both MTP8-GFP fusion proteins in onion cells resulted in MTP8-GFP proteins co-localizing with the Golgi marker GONST1, thus supporting localization in the Golgi apparatus (Fig. 3A and 3B). This suggests a physiological role for MTP8 proteins *in planta*, where they may load Mn into Golgi in order to either provide Mn for Mn dependent enzymes within the Golgi or to efflux Mn via exocytosis of secretory vesicles. This is in contrast to the rice MTP8 homolog shown to be localized in the vacuole with a suggested role in Mn detoxification based on vacuolar Mn sequestration [28].

To determine if MTP8 proteins have a role in Mn toxicity tolerance in barley, similar to the heterologous yeast system, expression analysis by RT-qPCR was performed. The gene expression data showed that plants exposed to excessive Mn supply resulted in down-regulation of both *MTP8* transcripts in leaf tissues (Fig. 6), as well as *MTP8.2* transcripts in roots (Fig. 6). This indicates that the primary physiological roles of MTP8 proteins are not directly related to detoxification of excess Mn *in planta*. Instead they may be involved in the delivery of Mn to metabolic processes of the Golgi.

In contrast, the expression pattern of *MTP8.1* in roots was positively correlated with the Mn concentration outside the root. Therefore, MTP8.1 may serve dual roles *in planta* both balancing excess Mn in roots and maintaining Mn homeostasis in Golgi in leaves. The *MTP8.1* isoform was highly represented among the yeast clones complementing the *pmr1* yeast strain in the original screen of the barley cDNA library, probably due to the generally higher transcript levels of MTP8.1 when compared to MTP8.2 in control plants (data not shown).

Hemicelluloses and pectins are synthesized in Golgi and require several different types of transport proteins to deliver sugars, acetyl-CoA, co-factors and ions to the working glycosyltransferases in the Golgi lumen [56]. Some glycosyltransferases require Mn for their activity [8]-[11] and due to their position in the Golgi lumen they are dependent on Mn-transporters placed in the Golgi membrane to facilitate metal delivery. The importance of the Golgi Mn pool may be directly visualized during Mn deficiency in plants, where slack leaves are a prominent and well described symptom. This is likely to be a result of Mn-deficiency induced defects in the delivery of intact cell wall components to growing cells.

Recent studies in mammalian cells indicate that the maintenance of Mn homeostasis is more complex than simply a matter of compartmentalization of Mn. A human Mn sensing glycoprotein (GPP130) has been found to induce vesicle trafficking from Golgi to multivesicular vesicles in response to elevated Golgi Mn concentrations as a way to regulate Mn extrusion from cells [57], [58]. A similar mechanism remains yet to be identified in plants.

## Conclusions

Several enzymes activated or stimulated by Mn have been isolated from the Golgi apparatus, but it has until now not been clear how Mn is transported into Golgi in monocot species. We have here isolated and characterised two MTP8 proteins, MTP8.1 and MTP8.2, and have shown that they localize to the Golgi apparatus when expressed in onion epidermal cells and function as Mn transporters when expressed in yeast. In barley roots, the expression of *MTP8.1* and *MTP8.2* responds differently to increasing Mn status, suggesting that the two genes undertake contrasting physiological functions with respect to control of Mn homeostasis. Further studies including barley lines with altered expression levels of the two genes are required for further clarification of the exact roles of the MTP8.1 and MTP8.2 proteins *in planta*.

## Supporting Information

S1 Figure **Alignment of the putative amino acid sequences of barley MTP's to other plant MTP proteins.** CLUSTAL W alignment of MTP proteins from *Arabidopsis thaliana*, *Oryza sativa*, *Stylosanthes hamate* and *Hordeum vulgare* was carried out using T-COFFEE (http://www.ch.embnet.org/software/TCoffee.html). The highly conserved N-terminal CDF signature sequence is marked with a box as well as the conserved aspartate and glycine residues are indicated with # and *, respectively.
(TIF)

S2 Figure **Effect of** ***MTP8.1*** **and** ***MTP8.2*** **expression on Mn, Fe, Zn and Cu accumulation in the yeast mutant** ***pmr1Δ*** **.** Mn, Fe, Zn and Cu accumulation in *pmr1Δ* yeast determined by ICP-OES analysis, cells were cultivated for 17 h with either 2.4 µM (black bars) or 100 µM (grey bars) Mn^2+^ in medium. Data are the means ± SE of three independent yeast samples. Values with the same letter within the same treatment are not significantly different (P>0.05).
(TIF)
