# Supplementary figures and images for: Golgi Localized Barley MTP8 Proteins Facilitate Mn Transport
Source: PLoS One. 2014 Dec 8;9(12):e113759. doi: 10.1371/journal.pone.0113759 (PMC4259309; doi:10.1371/journal.pone.0113759)

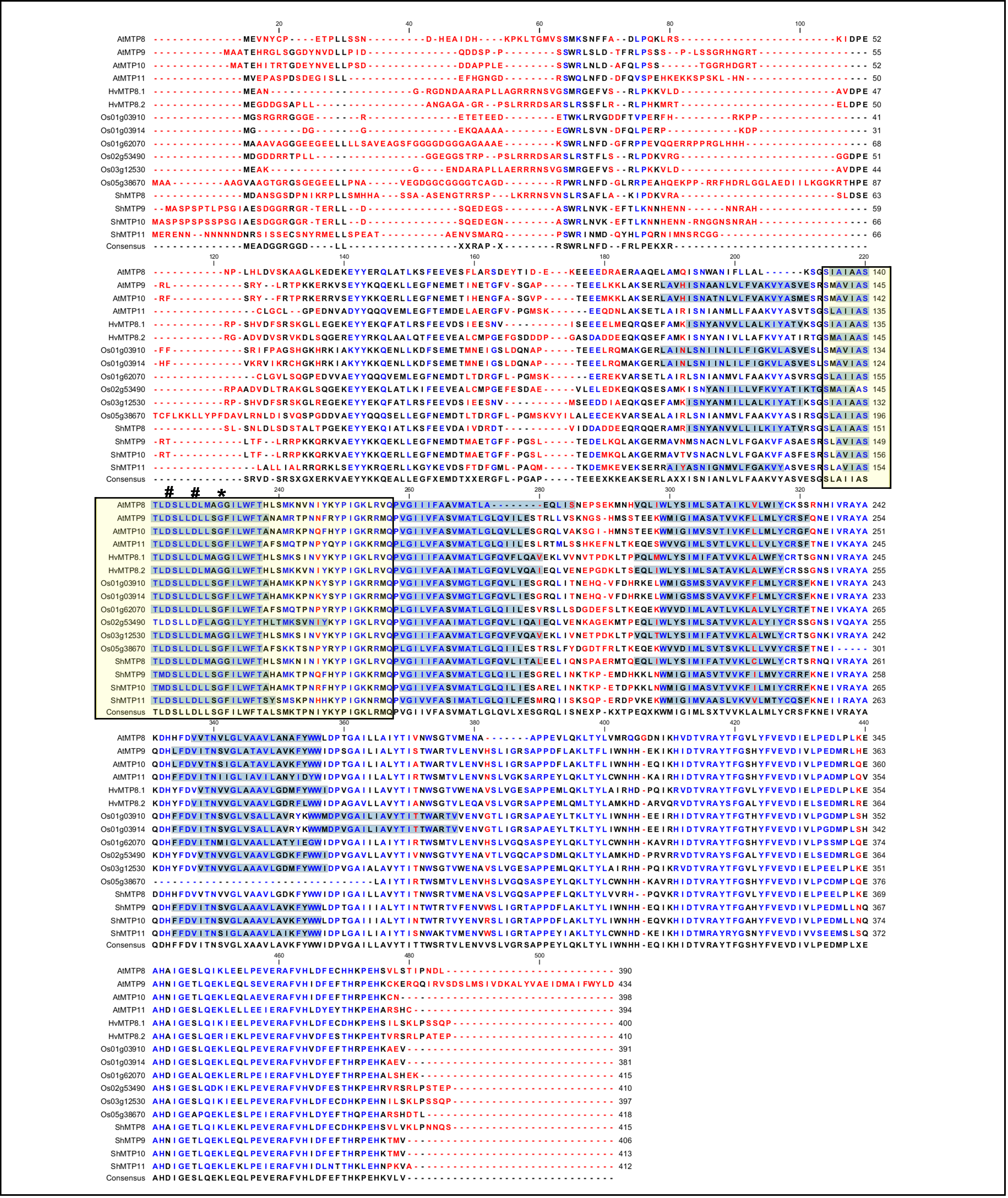

Supplement: S1 Figure — Alignment of the putative amino acid sequences of barley MTP's to other plant MTP proteins. CLUSTAL W alignment of MTP proteins from Arabidopsis thaliana, Oryza sativa, Stylosanthes hamate and Hordeum vulgare was carried out using T-COFFEE (http://www.ch.embnet.org/software/TCoffee.html). The highly conserved N-terminal CDF signature sequence is marked with a box as well as the conserved aspartate and glycine residues are indicated with # and *, respectively. (TIF) [file pone.0113759.s001.tif]
